# Correlation Between ^18^F-FDG Uptake and Immune Cell Infiltration in Metastatic Brain Lesions

**DOI:** 10.3389/fonc.2021.618705

**Published:** 2021-06-24

**Authors:** Young-Sil An, Se-Hyuk Kim, Tae Hoon Roh, So Hyun Park, Tae-Gyu Kim, Jang-Hee Kim

**Affiliations:** ^1^ Department of Nuclear Medicine and Molecular Imaging, Ajou University School of Medicine, Suwon, South Korea; ^2^ Department of Neurosurgery, Ajou University School of Medicine, Suwon, South Korea; ^3^ Department of Pathology, Ajou University School of Medicine, Suwon, South Korea

**Keywords:** ^18^F-fluorodeoxyglucose, positron emission tomography, brain metastasis, tumor microenvironment, immune cell

## Abstract

**Background:**

The purpose of this study was to investigate the correlation between ^18^F-fluorodeoxyglucose (FDG) uptake and infiltrating immune cells in metastatic brain lesions.

**Methods:**

This retrospective study included 34 patients with metastatic brain lesions who underwent brain ^18^F-FDG positron emission tomography (PET)/computed tomography (CT) followed by surgery. ^18^F-FDG uptake ratio was calculated by dividing the standardized uptake value (SUV) of the metastatic brain lesion by the contralateral normal white matter uptake value. We investigated the clinicopathological characteristics of the patients and analyzed the correlation between ^18^F-FDG uptake and infiltration of various immune cells. In addition, we evaluated immune-expression levels of glucose transporter 1 (GLUT1), hexokinase 2 (HK2), and Ki-67 in metastatic brain lesions.

**Results:**

The degree of ^18^F-FDG uptake of metastatic brain lesions was not significantly correlated with clinical parameters. There was no significant relationship between the ^18^F-FDG uptake and degree of immune cell infiltration in brain metastasis. Furthermore, other markers, such as GLUT1, HK2, and Ki-67, were not correlated with degree of ^18^F-FDG uptake. In metastatic brain lesions that originated from breast cancer, a higher degree of ^18^F-FDG uptake was observed in those with high expression of CD68.

**Conclusions:**

In metastatic brain lesions, the degree of ^18^F-FDG uptake was not significantly associated with infiltration of immune cells. The ^18^F-FDG uptake of metastatic brain lesions from breast cancer, however, might be associated with macrophage activity.

## Introduction

Brain metastasis is a serious clinical manifestation in cancer patients and develops in approximately 20–30% of patients with solid cancers (1–3). Although management of brain metastasis has improved with multimodal therapies, effective management remains a challenge and the outcome of brain metastases is uniformly poor, with less than 10% of patients with brain metastasis surviving more than 2 years (2, 4). Cancer immunotherapies, i.e. immune-checkpoint inhibitors (ICIs), have enhanced the overall survival of cancer patients and dramatically changed therapeutic strategies for metastatic and other advanced stage of certain types of cancers (5–7). Furthermore, clinical trials have provided evidence that ICIs or ICI combined with radiation therapy could have sustained treatment efficacy for brain metastases (1, 2). However, these treatments can increase the risk of adverse effects, i.e. neurologic toxicity or radiation necrosis, in patients with brain metastases (1, 8). Moreover, no definitive biomarkers have been identified that can differentiate patients with brain metastases who may benefit from ICIs from those at risk for adverse effects (8).

^18^F-Fluorodeoxyglucose (FDG) positron emission tomography (PET) is one of the fundamental imaging modalities for pre-therapeutic and therapeutic evaluation as well as end-of-treatment evaluations in clinical practice of many cancers (9–11). ^18^F-FDG uptake is associated with elevated glycolysis in cancer cells. However, ^18^F-FDG uptake can also be related to inflammation or immune reactions due to the consumption of glucose by immune cells (9, 11–13). Thus, ^18^F-FDG uptake in cancer can reflect the tumor microenvironment, including not only the metabolic activity of cancer cells but also local immune reactions (11, 14–16). Since the response to immunotherapy can be associated with tumor infiltrating immune cells (17–20) and immune cell response can be visualized by ^18^F-FDG PET (11, 14, 16), research has been conducted on the relationship between ^18^F-FDG uptake and immunological features of the tumor microenvironment. In certain types of primary cancers, ^18^F-FDG uptake is an additional biomarker that is predictive of immunological features and responses to ICIs (15, 21–24).

Since the microenvironment in brain metastases is different from the primary tumor (2, 25), to improve the efficiency of immunotherapy in brain metastases, a better understanding of the microenvironment in brain metastases, especially immune cell infiltration, is mandatory. However, few studies have used ^18^F-FDG uptake for evaluation of immune cell infiltrate in brain metastases (26). Therefore, here we investigated the correlation between ^18^F-FDG uptake and infiltration of various immune cells in brain metastases.

## Materials and Methods

### Subjects

This study included 34 patients who underwent brain ^18^F-FDG PET/computed tomography (CT) and were diagnosed with brain metastases at our institution between July 2005 and June 2019 with available brain tissue from surgery. We obtained clinical information (age, sex, primary cancer site, number of metastatic lesions in the brain, presence of metastatic lesions in regions other than brain and histologic type of metastatic brain lesion) from review of patient charts. This study was conducted retrospectively and was approved by the Institutional Review Board of Ajou University (AJIRB-MED-MDB-19-244). The need for informed consent was waived.

### Brain ^18^F-FDG PET/CT acquisition

After fasting for at least 6 h, patients were intravenously administered 300 MBq ^18^F-FDG. The blood glucose level at the time of the ^18^F-FDG injection was < 150 mg/dl in all patients. All subjects were instructed to rest comfortably for 30 min with their eyes closed before image acquisition. Brain PET/CT images were obtained with a Discovery ST 8 slice CT scanner or Discovery STE 16-slice CT scanner (GE Healthcare, Milwaukee, WI, USA). We first performed the non-contrast CT scan (100 kV, 95 mA; section width = 3.75 mm) in the brain region. Next, 10 min per frame of emission brain PET data were acquired in the three-dimensional mode. PET images were obtained by iterative reconstruction (i.e. ordered subsets of expectation maximization, with 2 iterations and 21 subsets), using CT images to correct attenuation.

### Quantitative Analysis of PET Data

**Figure 1:**
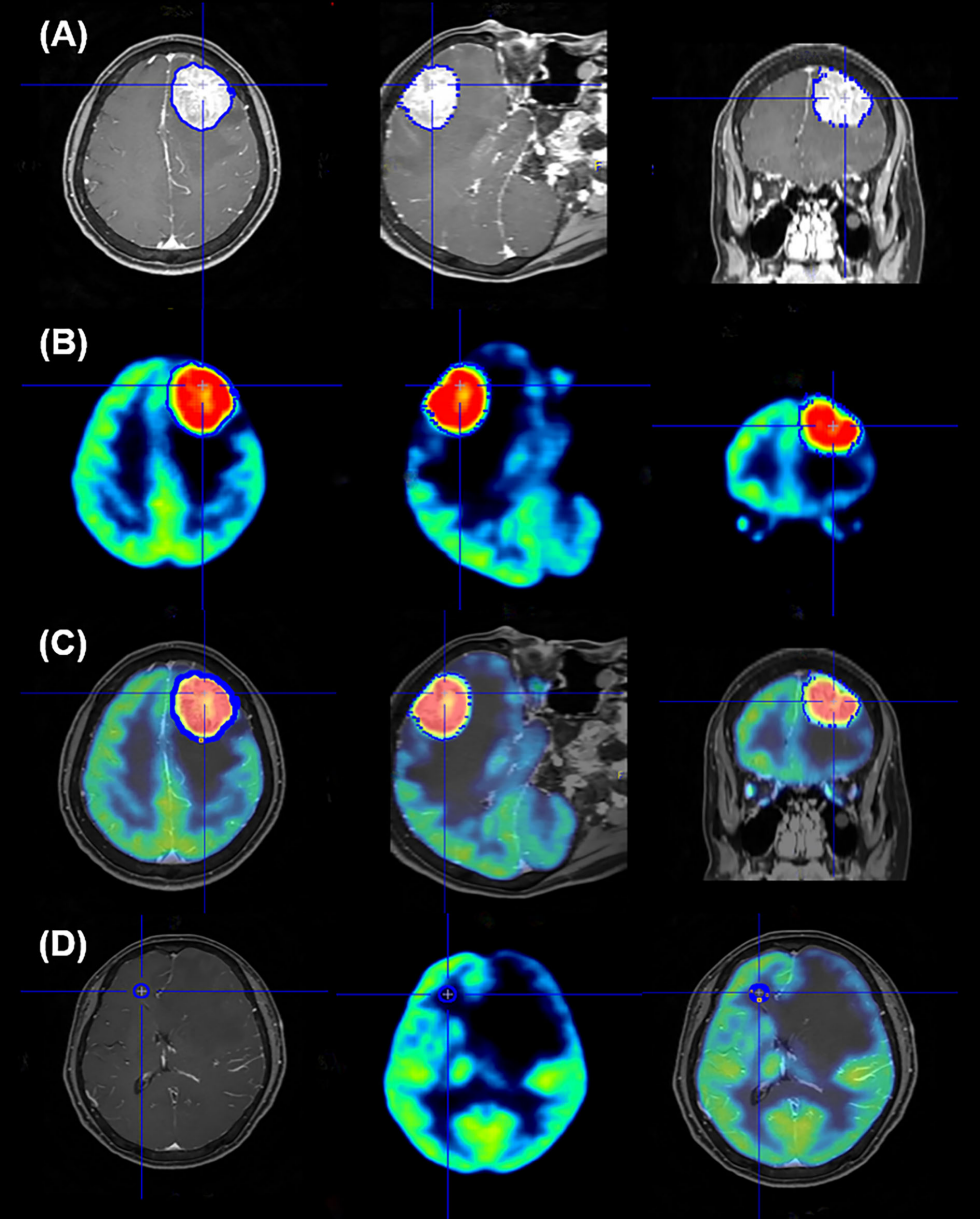
Representative image of region of interest setting for quantification analysis of ^18^F-FDG PET data. The volume of interest (VOI) was automatically delineated to brain lesions on MRI **(A)**; this edge of VOI was projected onto the PET image **(B)** and the VOI is seen in the image of the PET and MR fusion **(C)**. To set the reference area, the ROI is confirmed by setting the circular shape on the frontal white matter on the opposite side of the metastatic brain lesion and projecting it on the PET and PET/MR fusion images **(D)**.

After fusion of the gadolinium-enhanced T1-weighted magnetic resonance imaging (MRI) and brain ^18^F-FDG PET using the Fusion tool provided by PMOD software 3.0 (PMOD Technologies Ltd., Zurich, Switzerland), the volume of interest (VOI) was established by automatic delineation of the enhancing brain metastases lesions on MRI, which were removed by surgery. Edematous or necrotic areas of metastatic lesions, which could show considerably lower ^18^F-FDG accumulation, were excluded from VOI. The VOI set for MRI was projected on the PET image, and the maximum and mean standardized uptake values normalized for body weight (SUVmax and SUVmean, respectively) of VOI were recorded (**Figures 1A–C**). All images were visually assessed for correct co-registration and appropriate VOIs that did not include adjacent normal brain activity. To set the reference value, a circular region of interest (ROI) with a 10mm diameter was circularly drawn on the frontal white matter area of the contralateral brain without any abnormal findings on MRI based on previous studies (27–29), and the SUVmean values were obtained (**Figure 1D**). The ^18^F-FDG uptake ratio was calculated as the SUVs of the metastatic lesion divided by the SUVmean of the reference area. If multiple brain metastases were found in one patient, the lesion from which histological specimens were obtained was selected. We also measured the size of metastatic lesion, which had been excised and pathologically confirmed, on MRI images.

### Histopathologic Analysis and Interpretation

Immunohistochemistry was conducted on representative sections of formalin-fixed, paraffin-embedded tissues using a BenchMark XT automated immunohistochemistry stainer (Ventana Medical Systems, Inc., Tucson, AZ, USA) according to the manufacturer’s instructions. Briefly, after deparaffinization and rehydration, paraffin-embedded tissue sections (4-µm thick) were blocked with 3% hydrogen peroxide for 4 min at room temperature, treated with heat-induced antigen retrieval CC1 solution (Ventana Medical Systems) using the optimized antigen retrial condition, and incubated with primary antibodies. The primary antibodies are as follows: CD3, 1:100 (103R-95-RUO, Cell Marque, Rocklin, CA); CD8, pre-dilution (790-4460, Roche, Tucson, AZ); CD68, 1:50 (M0814, Dako, Denmark); CD163, 1:40 (163M-15-RUO, Cell Marque); myeloperoxidase (MPO), 1:100 (289A-75, Cell Marque); glucose transporter 1 (GLUT1), 1:200 (355A-14, Cell Marque); hexokinase 2 (HK2), 1:200 (E-AB-14706, Elabscience, Houston, TX) and Ki-67 (clone MIB-1) 1:60 (M7240, Dako). Detection was performed using the Ventana Optiview DAB Kit (Ventana Medical Systems). Counterstaining was performed with hematoxylin and bluing reagent for 4 min.

**Figure 2:**
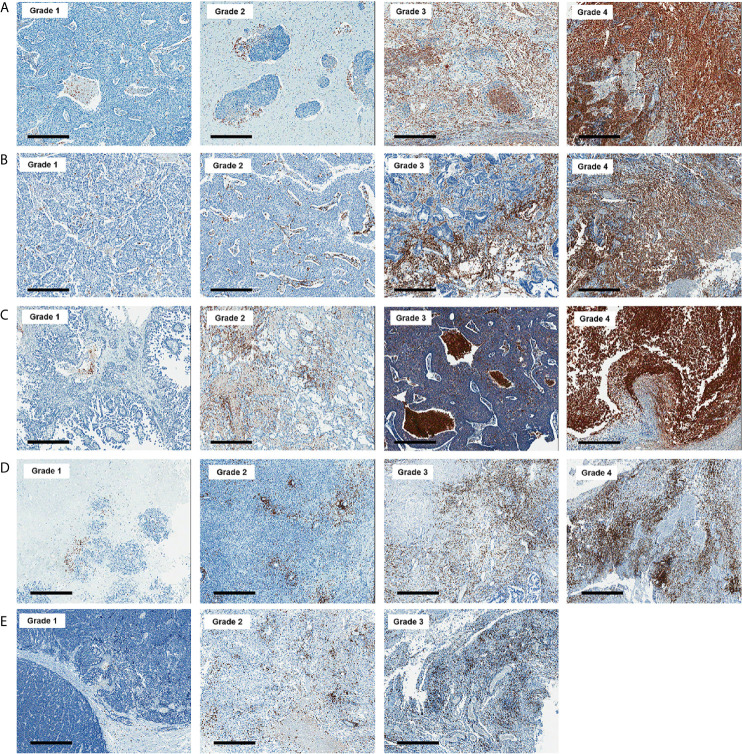
Representative images of CD68 **(A)**, CD163 **(B)**, myeloperoxidase **(C)**, CD3 **(D)**, and CD8 **(E)** immunohistochemistry according to grades (x 50). Bar indicates 500 μm.

**Figure 3:**
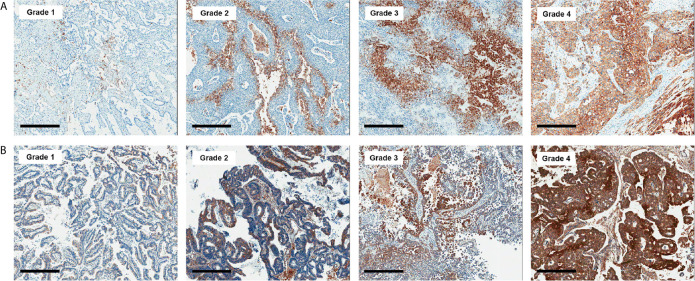
Representative images of GLUT1 **(A)** and hexokinase 2 **(B)** immunohistochemistry according to grades (x 50). Bar indicates 500 μm.

All histologic and immunohistochemical slides were reviewed by a single experienced pathologist (JH Kim) without prior knowledge of the clinical data and PET findings. Protein expression was evaluated based on intensity and proportion of positive cells. The intensity of expression was considered as positive if the intensity of membranous (GLUT1, CD3, and CD8), cytoplasmic (HK2, CD68, and CD163) or nuclear (Ki-67) staining was moderate or strong. Weak or nonspecific staining was considered as negative. For immune cell markers, we used CD3 for T cells, CD68 for macrophages, and MPO for neutrophils and eosinophils. Infiltration of immune cells was scored as follows: Grade 1, focal mild infiltration of positive cells; Grade 2, multifocal mild infiltration; Grade 3, multifocal moderate to marked infiltration; and Grade 4, diffuse moderate to marked infiltration (**Figure 2**). GLUT1 and HK2 expressions were scored based on the percentage of positive tumor cells as follows: Grade 1, positive tumor cells <10%; Grade 2, 10¬–40%; Grade 3, 40–70%; and Grade 4, >70% (**Figure 3**). The Ki-67 proliferation index was measured by counting the percentage of Ki-67-positive nuclei per 500–1000 tumor cells in the region of the tumor with the greatest density of staining, indicating areas with the highest mitotic activity.

### Statistical Analysis

The sample size required for this study using a significance (a) level of 5% and statistical power (1-b) of 80% was calculated using MedCalc software (version 18.11.3; MedCalc Software bvba, Ostend, Belgium). A sample size of 28 was required to obtain an appropriate confidence level; thus, the final sample size (n = 34) was sufficient.

Clinical characteristics are described as descriptive frequencies followed by percentages for categorical variables and means ± standard deviation (SD) for continuous variables. The difference in ^18^F-FDG uptake of brain metastatic lesions according to clinical characteristics was analyzed using Mann–Whitney test and one-way analysis of variance (ANOVA) test. Mann–Whitney or ANOVA test was used to determine whether ^18^F-FDG uptake varied according to the expression level of immune cell markers and biologic markers in metastatic brain lesions. Spearman’s correlation coefficient (r) was calculated to evaluate the correlations parameters. Correlations were classified as poor (│rho│< 0.29), fair (│rho│= 0.30–0.59), moderate (│rho│= 0.60–0.79), and very strong (│rho│≥ 0.80) (30). If there was a significant correlation between pathologic parameters, the analysis of covariance (ANCOVA) test was used to adjust the covariates. All other statistics were analyzed using MedCalc software. P-values <0.05 were considered significant.

## Results

### Patient Characteristics and ^18^F-FDG Uptake Ratio of Metastatic Brain Lesions

The average patient age was 63.7 years, and the patient group included 59% (20/34) males. Lung cancer (14/34, 41.2%) was the most common primary cancer site of metastatic brain lesions, followed by breast cancer (10/34, 29.4%). Twenty-one patients (21/34, 61.8%) had a single metastatic brain lesion. Approximately 44% of patients (15/34) also had metastases to other organs at the time of diagnosis of brain metastasis. Most of the histologic types of the metastatic lesions were adenocarcinoma (29/34, 85.4%). The mean diameter and VOI of brain metastases lesions were 3.10 cm and 13.08 cm^3^, respectively. The mean value of maximum ^18^F-FDG uptake ratio and of brain metastases was 3.02, and the average value of the mean ^18^F-FDG uptake ratio was 1.70. The degree of ^18^F-FDG uptake of brain metastasis lesions showed poor correlation with lesion size and VOI size (all *p* > 0.05) (**Table 1**). The mean and maximum ^18^F-FDG uptake ratios were slightly lower in the metastatic brain lesions from the lung than those in other sites. However, the difference was not statistically significant. The other clinicopathological parameters, i.e. histologic type of the metastatic lesions, showed no significant correlation (all *p* > 0.05). **Table 1** lists the clinicopathological characteristics of patients and the difference in ^18^F-FDG uptake of brain metastasis according to clinical parameters. The individual characteristics of each patient are presented in **Supplementary Table 1**.

**Table 1:** Clinicopathologic characteristics and ^18^F-FDG uptake ratio in patients with brain metastases.

Characteristics	Number	Maximum ^18^F-FDG uptake ratio	*p*-value for difference of maximum ^18^F-FDG uptake ratio between groups	Mean ^18^F-FDG uptake ratio	*p*-value for difference of mean ^18^F-FDG uptake ratio between groups
Age (years)	63.70 ± 9.90	3.02 ± 1.24	NA	1.70 ± 0.70	NA
Sex					
Male	20 (58.8%)	3.08 ± 1.39	0.743	1.58 ± 0.65	0.261
Female	14 (41.2%)	2.93 ± 1.05		1.86 ± 0.76	
Primary cancer sites					
Lung	14 (41.2%)	2.74 ± 0.95	0.547	1.51 ± 0.58	0.273
Breast	10 (29.4%)	3.12 ± 1.04		1.98 ± 0.82	
GI tract and others	10 (29.4%)	3.29 ± 1.75		1.67 ± 0.70	
Number of metastatic sites in the brain					
Single	21 (61.8%)	2.87 ± 0.96	0.375	1.64 ± 0.68	0.530
Multiple	13 (38.2%)	3.28 ± 1.66		1.80 ± 0.75	
Presence of extracranial metastasis					
Yes	15 (44.1%)	3.35 ± 1.42	0.165	1.73 ± 0.78	0.756
No	19 (55.9%)	2.75 ± 1.05		1.65 ± 0.60	
Histologic type of metastatic lesions					
Adenocarcinoma^†^	29 (85.4%)	3.08 ± 1.28	0.371	1.71 ± 0.69	0.118
Squamous cell carcinoma	3 (8.8%)	2.03 ± 0.16		1.17 ± 0.25	
Small cell carcinoma	1 (2.9%)	2.67		1.52	
Large cell neuroendocrine carcinoma	1 (2.9%)	4.38		3.11	
		**Correlation with maximum ^18^F-FDG uptake ratio**		**Correlation with mean ^18^F-FDG uptake ratio**	
		**rho (95% CI)**	***p*** **-value**	**rho (95% CI)**	***p*-value**
Size of brain metastasis (cm)	3.10 ± 0.94	0.27 (0.07 to 0.56)	0.114	0.03 (-0.31 to 0.36)	0.879
VOI size of brain metastasis (cm^3^)	13.08 ± 9.94	0.23 (0.11 to 0.53)	0.175	0.02 (-0.35 to 0.32)	0.896

^†^Category of adenocarcinoma included adenocarcinoma of the lung and gastrointestinal tracts, as well as ductal and lobular carcinoma of the breast.

GI tract, Gastrointestinal tract; NA, Not available.

### Relationship Between ^18^F-FDG Uptake and Grades of Immune Cell Infiltration

We next identified immune cells using specific markers. Macrophages, which are immune-positive for CD68 and/or CD163, were most abundantly identified in the metastatic brain lesions, followed by neutrophils, which are immune-positive for MPO. Diffuse strong infiltration (grade 4) of CD163+ macrophages was observed in 5 (14.7%) of metastatic lesions. Two (5.9%) cases showed diffuse strong infiltration of neutrophils, and both cases were associated with tumor necrosis. However, T lymphocytes in metastatic brain lesions were less frequently identified and only one case revealed diffuse strong infiltration (Grade 4) of CD3+ T lymphocytes. Moreover, most cases (27/34, 79.4%) showed only focal mild infiltration or no diffuse strong infiltration of CD8+ T lymphocytes (**Table 2**). We performed analysis of ^18^F-FDG uptake ratio of brain metastases according to the grades of infiltration of each immune cell. To evaluate positive or negative effects between types of immune cells, we analyzed correlation between grades of immune markers. We observed a significantly positive correlation between markers for macrophages (CD68 and CD163) and T cells (CD3 and CD8). However, we could not find any significant differences between ^18^F-FDG uptake ratio and grades of immune cell infiltration with or without adjustment for covariates (all *p* > 0.05) (**Table 2** and **Supplementary Table 2**).

**Table 2:** ^18^F-FDG uptake ratio according to GLUT1, HK2 and immune cell markers in patients with brain metastasis.

	Maximum ^18^F-FDG uptake ratio	*p*-value for difference of maximum ^18^F-FDG uptake ratio between groups	Mean ^18^F-FDG uptake ratio	*p*-value for difference of mean ^18^F-FDG uptake ratio between groups
Expression of CD68
Grade 1 (*n*=13)	3.02 ± 1.54		1.62 ± 0.70
Grade 2 (*n*=10)	3.35 ± 1.09	0.384^†^	1.98 ± 0.77	0.216^†^
Grade 3 (*n*=6)	2.63 ± 1.09		1.36 ± 0.44
Grade 4 (*n*=5)	2.80 ± 0.98		1.72 ± 0.79
Expression of CD163		
Grade 1 (*n*=9)	3.21± 1.74		1.63 ± 0.77	
Grade 2 (*n*=12)	3.24± 1.10	0.865^†^	1.93 ± 0.72	0.748^†^
Grade 3 (*n*=8)	2.74 ± 1.00		1.40 ± 0.49	
Grade 4 (*n*=5)	2.58 ± 1.01		1.71 ± 0.79	
Expression of MPO				
Grade 1 (*n*=13)	3.46 ± 1.59		2.03 ± 0.83	
Grade 2 (*n*=10)	2.44 ± 0.68	0.271^†^	1.42 ± 0.38	0.074^†^
Grade 3 (*n*=9)	3.23 ± 1.01		1.66 ± 0.66	
Grade 4 (*n*=2)	2.02 ± 0.07		1.09 ± 0.12	
Expression of CD3				
Grade 1 (*n*=18)	3.25 ± 1.39		1.78 ± 0.74	
Grade 2 (*n*=14)	2.85 ± 1.06	0.350^†^	1.64 ± 0.69	0.279^†^
Grade 3 (*n*=1)	2.02		1.40	
Grade 4 (*n*=1)	2.05		1.26	
Expression of CD8				
Grade 1 (*n*=27)	3.01 ± 1.29		1.68 ± 0.68	
Grade 2 (*n*=5)	3.47 ± 1.06	0.312^†^	1.93 ± 0.92	0.529^†^
Grade 3 (*n*=2)	2.03 ± 0.02		1.33 ± 0.09	

^†^adjusted values for covariates; MPO, Myeloperoxidase, marker for neutrophils; CD3/CD8, Marker for T cells; CD68/CD163, Marker for macrophages.

### Relationship Between ^18^F-FDG Uptake and Grades of Immune Cell Infiltration According to the Primary Cancer Sites

We further investigated immune cell infiltration according to the sites of the primary cancers. We divided metastatic brain lesions into 3 groups (lung [*n*=14], breast [*n*=10], and GI and others [*n*=10]) according to the primary sites and analyzed immune cell infiltration in each group. Immune cell infiltration was more frequent in the metastatic lesions from the lung than the other two groups (**Table 3**). In particular, diffuse strong infiltration of macrophages (CD68 or CD163) was most commonly identified in the metastatic lesion from the lung. In the majority of metastatic lesions from the breast, immune cell infiltrations except for neutrophils (MPO) were mild (Grade 1 or 2). We analyzed the correlation between ^18^F-FDG uptake ratio of brain metastases lesions and primary cancer sites. Interestingly, we found that the maximum ^18^F-FDG uptake ratio and mean ^18^F-FDG uptake ratio were significantly correlated with infiltration of macrophages (CD68) in the metastatic lesions with breast origin (*p* = 0.002 and *p* =0.036, respectively). There were no associations between ^18^F-FDG uptake ratios and infiltration of other types of immune cells (**Table 3**).

**Table 3:** ^18^F-FDG uptake ratio and expression of immune cell markers according to the primary cancer.

Primary cancer	Immune cell markers	Maximum ^18^F-FDG uptake ratio	*p*-value for difference of maximum ^18^F-FDG uptake ratio between groups	Mean ^18^F-FDG uptake ratio	*p*-value for difference of mean^18^F-FDG uptake ratio between groups
Lung	Expression of CD68				
	Grade 1 (*n*=4)	3.03 ± 1.04		1.64 ± 0.51	
	Grade 2 (*n*=2)	2.28 ± 0.61	0.909^†^	1.24 ± 0.45	0.688^†^
	Grade 3 (*n*=3)	2.56 ± 1.31		1.17 ± 0.29	
	Grade 4 (*n*=5)	2.80 ± 0.98		1.72 ± 0.79	
	Expression of CD163				
	Grade 1 (*n*=2)	3.19 ± 0.74		1.56 ± 0.06	
	Grade 2 (*n*=3)	2.53 ± 1.28	0.764^†^	1.45 ± 0.77	0.632^†^
	Grade 3 (*n*=4)	2.88 ± 1.02		1.27 ± 0.31	
	Grade 4 (*n*=5)	2.58 ± 1.01		1.71 ± 0.79	
	Expression of MPO				
	Grade 1 (*n*=3)	2.80 ± 1.14		1.65 ± 0.63	
	Grade 2 (*n*=5)	2.47 ± 0.77	0.722^†^	1.35 ± 0.27	0.767^†^
	Grade 3 (*n*=5)	3.11 ± 1.15		1.65 ± 0.86	
	Grade 4 (*n*=1)	2.08		1.18	
	Expression of CD3				
	Grade 1 (*n*=5)	2.79 ± 1.04		1.49 ± 0.55	
	Grade 2 (*n*=7)	2.91 ± 1.02	0.253^†^	1.57 ± 0.72	0.452^†^
	Grade 3 (*n*=1)	2.02		1.40	
	Grade 4 (*n*=1)	2.05		1.26	
	Expression of CD8				
	Grade 1 (*n*=9)	2.57 ± 0.88		1.39 ± 0.45	
	Grade 2 (*n*=3)	3.72 ± 0.88	0.190^†^	1.98 ± 0.98	0.307^†^
	Grade 3 (*n*=2)	2.03 ± 0.02		1.33 ± 0.09	
Breast	Expression of CD68				
	Grade 1 (*n*=6)	2.36 ± 0.40^*^		1.47 ± 0.60^*^	
	Grade 2 (*n*=4)	4.27 ± 0.28^*^	0.002^†*^	2.75 ± 0.29^*^	0.036^†*^
	Expression of CD163				
	Grade 1 (*n*=4)	2.39 ± 0.42		1.45 ± 0.73	
	Grade 2 (*n*=6)	3.61 ± 1.06	0.818^†^	2.33 ± 0.72	0.927^†^
	Expression of MPO				
	Grade 1 (*n*=8)	3.37 ± 1.01		2.08 ± 0.87	
	Grade 2 (*n*=1)	2.32	0.348	2.03	0.628
	Grade 3 (*n*=1)	1.95		1.16	
	Expression of CD3				
	Grade 1 (*n*=7)	3.02 ± 1.03		1.94 ± 0.88	
	Grade 2 (*n*=3)	3.37 ± 1.23	0.659	2.08 ± 0.81	0.827
	Expression of CD8				
	Grade 1 (*n*=9)	3.00 ± 1.02		1.90 ± 0.82	
	Grade 2 (*n*=1)	4.22	0.295	2.72	0.377
GI tract and others	Expression of CD68				
	Grade 1 (*n*=3)	4.32 ± 2.90		1.92 ± 1.18	
	Grade 2 (*n*=4)	2.97 ± 1.13	0.974^†^	1.58 ± 0.49	0.980^†^
	Grade 3 (*n*=3)	2.71 ± 1.11		1.55 ± 0.54	
	Expression of CD163				
	Grade 1 (*n*=3)	4.32 ± 2.90		1.92 ± 1.18	
	Grade 2 (*n*=3)	3.19 ± 1.03	0.967^†^	1.61 ± 1.11	0.970^†^
	Grade 3 (*n*=4)	2.61 ± 1.11		1.53 ± 0.66	
	Expression of MPO				
	Grade 1 (*n*=2)	4.82 ± 3.97		2.41 ± 1.22	
	Grade 2 (*n*=4)	2.45 ± 0.77	0.400	1.35 ± 0.46	0.262
	Grade 3 (*n*=3)	3.86 ± 0.16		1.84 ± 0.34	
	Grade 4 (*n*=1)	1.97		1.00	
	Expression of CD3				
	Grade 1 (*n*=6)	3.91 ± 1.91		1.84 ± 0.76	
	Grade 2 (*n*=4)	2.37 ± 1.11	0.487^†^	1.42 ± 0.59	0.594^†^
	Expression of CD8				
	Grade 1 (*n*=9)	3.44 ± 1.79		1.75 ± 0.69	
	Grade 2 (*n*=1)	1.97	0.459	1.00	0.262

^†^adjusted values for covariates, ^*^p < 0.05; GI tract: Gastrointestinal tract; MPO, Myeloperoxidase, marker for neutrophils; CD3/CD8, Marker for T cells; CD68/CD163, Marker for macrophages.

### Relationship Between ^18^F-FDG Uptake and Expression of Other Biologic Markers in the Metastatic Brain Lesions

**Figure 4:**
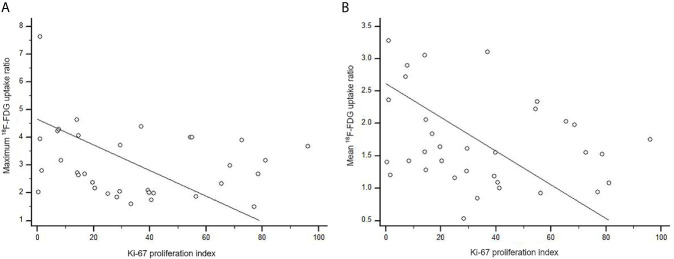
Scatter diagram of the correlation between Ki-67 proliferation index and ^18^F-FDG uptake ratio in metastatic brain lesion (total, n = 34). **(A)** The maximum ^18^F-FDG uptake ratio of brain metastatic lesions poorly correlated with Ki-67 proliferation index without statistical significance (rho = -0.21, p = 0.227). **(B)** There was no significant correlation between Ki-67 proliferation index and mean 18F-FDG uptake ratio (rho = -0.25, p = 0.143).

We also examined immuno-expression of GLUT1, HK2 and Ki-67, which are known biologic markers associated with ^18^F-FDG uptake. We found that 10 cases (29.4%) showed diffuse strong immuno-expression (grade 4) of HK2 in metastatic tumor cells and 7 cases (20.6%) revealed diffuse strong immuno-expression (grade 4) of GLUT1. However, the maximum ^18^F-FDG uptake ratio and mean ^18^F-FDG uptake ratio in metastatic brain lesions did not differ significantly according to the grades of GLUT1 and HK2 after adjustment for covariates (all *p* > 0.05) (**Table 4** and **Supplementary Table 2**). The absence of association between ^18^F-FDG uptake ratio and degrees of GLUT1 and HK2 expression also was found within subgroups divided by primary cancer site (**Table 4**). The Ki-67 proliferation index of the metastatic brain lesions ranged widely from 0.3% to 96.1% (average: 35.0%). However, the Ki-67 proliferation index of the metastatic lesions showed not only poor correlation but also inverse tendency with ^18^F-FDG uptake ratios (for maximum ^18^F-FDG uptake ratio, rho = -0.21; for mean ^18^F-FDG uptake ratio, rho = -0.25; **Figure 4** and **Table 5**). The Ki-67 proliferation index and ^18^F-FDG uptake ratio did not show a significant correlation even within the subgroups by primary cancer site (all *p* > 0.05, **Table 5**).

**Table 4:** ^18^F-FDG uptake ratio according to the expression of GLUT1 and HK2 in patients with brain metastasis.

Primary cancer		Maximum ^18^F-FDG uptake ratio	*p*-value for difference of maximum ^18^F-FDG uptake ratio between groups	Mean ^18^F-FDG uptake ratio	*p*-value for difference of mean ^18^F-FDG uptake ratio between groups
Total	Expression of GLUT1				
	Grade 1 (*n*=8)	3.26 ± 0.91		2.02 ± 0.69	
	Grade 2 (*n*=11)	2.85 ± 1.75	0.978^†^	1.49 ± 0.74	0.755^†^
	Grade 3 (*n*=8)	2.95 ± 0.89		1.63 ± 0.58	
	Grade 4 (*n*=7)	3.07 ± 1.18		1.70 ± 0.77	
	Expression of HK2				
	Grade 1 (*n*=6)	2.71 ± 0.93		1.60 ± 0.76	
	Grade 2 (*n*=9)	2.97 ± 0.87	0.641	1.72 ± 0.55	0.988
	Grade 3 (*n*=9)	2.80 ± 1.06		1.73 ± 0.65	
	Grade 4 (*n*=10)	3.44 ± 1.79		1.70 ± 0.90	
Lung	Expression of GLUT1				
	Grade 1 (*n*=2)	2.59 ± 0.81		1.41 ± 0.01	
	Grade 2 (*n*=4)	2.08 ± 0.21	0.410	1.25 ± 0.29	0.612
	Grade 3 (*n*=3)	3.15 ± 0.79		1.45 ± 0.15	
	Grade 4 (*n*=5)	3.08 ± 1.32		1.79 ± 0.93	
	Expression of HK2				
	Grade 1 (*n*=5)	2.86 ± 0.96		1.72 ± 0.78	
	Grade 2 (*n*=1)	1.85	0.733	0.92	0.307
	Grade 3 (*n*=5)	2.97 ± 0.85		1.68 ± 0.39	
	Grade 4 (*n*=3)	2.46 ± 1.38		1.09 ± 0.22	
Breast	Expression of GLUT1				
	Grade 1 (*n*=6)	3.49 ± 0.90		2.23 ± 0.69	
	Grade 2 (*n*=2)	1.89 ± 0.08	0.166	0.84 ± 0.44	0.067
	Grade 3 (*n*=2)	3.27 ± 1.34		2.37 ± 0.48	
	Expression of HK2				
	Grade 2 (*n*=2)	3.45 ± 1.08		2.28 ± 0.62	
	Grade 3 (*n*=3)	2.96 ± 1.45	0.900	2.08 ± 0.95	0.811
	Grade 4 (*n*=5)	3.09 ± 1.01		1.81 ± 0.94	
GI tract and others	Expression of GLUT1				
	Grade 2 (*n*=5)	3.86 ± 2.30			
	Grade 3 (*n*=3)	2.54 ± 0.91	0.631		0.483
	Grade 4 (*n*=2)	3.03 ± 1.21			
	Expression of HK2				
	Grade 1 (*n*=1)	1.97			
	Grade 2 (*n*=6)	3.00 ± 0.79	0.091		0.248
	Grade 3 (*n*=1)	1.49			
	Grade 4 (*n*=2)	5.76 ± 2.64			

^†^adjusted values for covariates; GLUT1, Glucose transporter 1; HK2, Hexokinase 2.

**Table 5:** Correlation between ^18^F-FDG uptake ratio and Ki-67 proliferation index in patients with brain metastasis.

Primary cancer	Ki-67 proliferation index (%)	Correlation with maximum ^18^F-FDG uptake ratio		Correlation with mean ^18^F-FDG uptake ratio	
		rho (95% CI)	*p*-value	rho (95% CI)	*p*-value
Total	35.0 ± 26.9	-0.21 (-0.56 to 0.06)	0.112	-0.25 (-0.57 to 0.05)	0.094
Lung	32.6 ± 21.1	-0.14 (-0.62 to 0.42)	0.637	-0.13 (-0.61 to 0.43)	0.670
Breast	18.2 ± 18.9	-0.27 (-0.49 to 0.16)	0.721	-0.18 (-0.75 to 0.20)	0.491
GI tract and others	55.3 ± 29.6	-0.24 (-0.66 to 0.09)	0.581	-0.26 (-0.77 to 0.38)	0.423

CI, Confidence Interval.

## Discussion

The tumor microenvironment of brain metastases is unique and distinct from other sites of the body not only in terms of cellular components but also in metabolism (25, 31, 32). The cellular components of brain include astrocytes, microglia, oligodendrocytes, and neurons that are not present elsewhere in the body (2, 25, 32). In addition, parenchymal cells of the normal brain show high levels of glucose metabolism (33) and this metabolic characteristic of normal brain hampers the delineation of tumors from normal brain by ^18^F-FDG compared with amino acid tracers, such as ^11^C-methionine (MET) and 6-[18F]-L-fluoro-L-3, 4-dihydroxyphenylalanine (FDOPA) (34). Nevertheless, ^18^F-FDG is clinically preferred because commercially available ^18^F-FDG is the easiest to - perform in facilities without cyclotrons and costs are mostly covered by health insurance, though this can vary from country to country, but also shows cost benefits. In addition, previous studies reported that ^18^F-FDG PET provides valuable information on the metabolic status of the tumor microenvironment as well as local immune reactions (14, 15, 21–24, 35). To overcome the weakness of ^18^F-FDG in the brain and enhance the delineation of tumor from normal brain, we defined the reference value; the ROI was circularly drawn on the frontal white matter area of the contralateral brain without any abnormal findings on MRI based on previous studies (27–29). We found a wide range of ^18^F-FDG uptake in metastatic brain lesions.

^18^F-FDG can be taken up by many tumor-associated immune cells, such as tumor-infiltrating lymphocytes (TILs), tumor-associated macrophages (TAMs) and granulocytes such as neutrophils (11, 14, 16). ^18^F-FDG uptake is correlated with PDL-1 expression and TILs, especially CD8+ cytotoxic T cells in primary cancers (14, 22–24). Furthermore, abundant infiltration of TILs including CD8+ T cells in primary cancers is associated with better response to immunotherapy (18, 20, 36, 37). Here we investigated the correlation between ^18^F-FDG uptake and immune cell infiltration with various immune cell markers in brain metastases. In contrast to previous reports with primary cancers (14, 22–24), there were no significant correlations between ^18^F-FDG uptake and T cell infiltration grade in brain metastases.

^18^F-FDG uptake of immune cells in brain metastases can differ from uptake in other sites of the body. Immune cells including lymphocytes, neutrophils and the monocyte/macrophage family express high levels of glucose transporters and hexokinase activity with increased ^18^F-FDG uptake (12, 13). However, immune cells in metastatic brain lesions compete to utilize glucose not only with tumor cells but also brain parenchymal cells, because brain parenchymal cells have also high glucose metabolism (33, 38). This difference of the metabolic environment in brain makes the mechanism of ^18^F-FDG uptake in brain metastases highly complex. We suggest the possibility that such a unique tumor microenvironment may be one of possible explanations of our negative results.

In brain metastases, T cell infiltration tends to be less frequent than in peripherally located primary lesions whereas infiltration of microglia and monocytes can be abundant (39–41). We found that among immune cells, macrophages most frequently showed diffuse strong infiltration in brain metastases (14.7%, 5/34 cases) followed by neutrophils (5.9%, 2/34 cases). We observed a significantly positive correlation between grades of macrophages markers (CD68 and CD163) and T cell markers (CD3 and CD8). Interestingly, infiltration of macrophages (CD68+) was significantly associated with increased ^18^F-FDG uptake in metastatic lesions from the breast, although the number of patients was as small at 10. Contrary to our expectation, the majority of metastatic lesions from the breast revealed only focal mild or multifocal mild infiltration of immune cell infiltration except for neutrophils. In addition, different from other types of metastatic tumors, infiltration of macrophages (CD68) in metastatic breast tumor showed no significant correlation with T cell markers but revealed a negative correlation with Ki-67 proliferative index, suggesting that a less complex immune environment and a low proliferation rate of tumor cells could explain the result. The association of ^18^F-FDG uptake with immune cell infiltration in primary breast cancers is controversial. Kajary et al. (42) reported no correlation between TILs and kinetic parameters using whole-body ^18^F-FDG PET. However, other studies revealed a significant correlation between ^18^F-FDG uptake and TILs in breast cancers (21, 22, 43). Furthermore, all of these studies have focused only on TILs in primary tumors and did not investigate other immune cells such as macrophages. In brain metastases, similar to brain tumors, the majority of immune cells are macrophages that may hinder the cell mediated immune response in metastasis (39–41). Macrophages can polarize as either M1 macrophages or M2 macrophages. M1 macrophages can produce inflammatory mediators directed against pathogens and tumor cells, while M2 macrophages are involved in immunosuppression and repair. Tumor associated macrophages (TAMs) take on a pro-tumoral M2 phenotype involved in growth, extracellular matrix remodeling, angiogenesis and immunosuppression (32, 39, 40, 44). However, such an oversimplification of macrophage phenotype has been disputed because the status of macrophage activation reveals a much wider range *in vivo* (32, 39). In addition to CD68, we also analyzed macrophages using CD163, which is one of the markers suggesting M2 macrophages. Infiltration of CD163+ macrophages was slightly higher than that of CD68+ macrophages. However, we could not find a significant correlation between the infiltration of CD163+ macrophages and ^18^F-FDG uptake.

Pukrop T et al. (45) reported that microglia/macrophages can be identified in brain metastases from the breast, ranging from only few to up to 50% of all cells. TAMs within the brain tend to be pro-tumorigenic and TAM depletion strategies may provide a survival advantage in several types of cancer (32). Activated TAMs promoted cancer cell invasion and colonization of the brain tissue *in vitro* whereas blocking microglia function reduced cancer cell invasion. However, TAMs can be activated by cancer cells without polarization to M2 macrophages (45). We found that infiltration of CD68+ macrophages in brain metastases was only significantly correlated with maximum and mean ^18^F-FDG uptake ratios at the metastatic lesions (p < 0.001 and p = 0.005, respectively). Since TAM density is associated with poor prognosis in breast cancer patients and eliminating macrophages from the tumor site in mouse models of breast cancer induced a delay of tumor progression, targeting TAM in brain metastases from the breast may provide a new therapeutic strategy (25, 40, 41, 46).

In addition to immune cell infiltration in brain metastases, we also analyzed additional biologic makers related to ^18^F-FDG uptake. ^18^F-FDG uptake in cancer tissues from primary malignant lesions is commonly associated with high levels of HK and GLUT (47–50). In addition, the Ki-67 proliferation index, which indicates the growth rate of tumor cells, is also associated with tumor ^18^F-FDG uptake (51). However, we could not find associations of ^18^F-FDG uptake in brain metastases with GLUT1 or HK2 or the Ki-67 proliferation index. We cannot explain these negative results. However, we suggest that the mechanism involving ^18^F-FDG uptake in the brain metastases may be more complex than in the primary cancer due to a unique tumor microenvironment in which not only cancer cells but also immune cells and brain parenchymal cells compete to utilize glucose for survival (25, 52). The complex mechanisms that influence ^18^F-FDG uptake in brain metastasis remain to be determined. Therefore, our study may provide reference data for subsequent studies to address the mechanism of ^18^F-FDG uptake in brain metastases.

The small number of patients included in this study may be a limitation to our study. It was not easy to find cancer patients available for brain ^18^F-FDG PET and with pathological data of metastatic brain lesion. Although we confirmed that the number of patients in this study satisfied the statistically meaningful sample size, we also acknowledge that the sample number is small. In particular, the number of samples in subgroups according to primary cancer site was very small. Therefore, future studies including large samples are needed to validate our study results.

In conclusion, we investigated the degree of ^18^F-FDG uptake in brain metastases and its correlation with immune cell infiltration and several biologic markers. ^18^F-FDG uptake was not correlated with immune cells and other biologic markers. However, in certain types of metastatic cancer, ^18^F-FDG uptake may be a non-invasive tool for predicting immunological features of brain metastases.

## Data Availability Statement

The raw data supporting the conclusions of this article will be made available by the authors, without undue reservation.

## Ethics Statement

This study was conducted retrospectively and was approved by the Institutional Review Board of Ajou University (AJIRB-MED-MDB-19-244). Written informed consent for participation was not required for this study in accordance with the national legislation and the institutional requirements.

## Author Contributions

Y-SA and J-HK conceived of the presented idea. Y-SA and J-HK developed the theory and performed the computations. S-HK, TR, SP, and T-GK verified the analytical methods. J-HK encouraged SP and T-GK to investigate histologic aspect and supervised the findings of this work. All authors contributed to the article and approved the submitted version.

## Funding

This work was supported by the faculty research fund of Ajou University School of Medicine to S-HK and J-HK, National Research Foundation of Korea to J-HK (NRF-2016R1D1A1B02010452), and Basic Science Research Program through the National Research Foundation of Korea (NRF) funded by the Ministry of Education (NRF-2020R1A6A1A03043539) to J-HK.

## Conflict of Interest

The authors declare that the research was conducted in the absence of any commercial or financial relationships that could be construed as a potential conflict of interest.

## References

[1] Suh JH Kotecha R Chao ST Ahluwalia MS Sahgal A Chang EL. Current Approaches to the Management of Brain Metastases. Nat Rev Clin Oncol (2020) 17(5):279–99. 10.1038/s41571-019-0320-3 32080373

[2] Achrol AS Rennert RC Anders C Soffietti R Ahluwalia MS Nayak L. Brain Metastases. Nat Rev Dis Primers (2019) 5(1):5. 10.1038/s41572-018-0055-y 30655533

[3] Cagney DN Martin AM Catalano PJ Redig AJ Lin NU Lee EQ. Incidence and Prognosis of Patients With Brain Metastases at Diagnosis of Systemic Malignancy: A Population-Based Study. Neuro Oncol (2017) 19(11):1511–21. 10.1093/neuonc/nox077 PMC5737512 28444227

[4] Hall WA Djalilian HR Nussbaum ES Cho KH. Long-Term Survival With Metastatic Cancer to the Brain. Med Oncol (2000) 17(4):279–86. 10.1007/BF02782192 11114706

[5] Gauci ML Lanoy E Champiat S Caramella C Ammari S Aspeslagh S. Long-Term Survival in Patients Responding to Anti-PD-1/PD-L1 Therapy and Disease Outcome Upon Treatment Discontinuation. Clin Cancer Res (2019) 25(3):946–56. 10.1158/1078-0432.CCR-18-0793 30297458

[6] Topalian SL Sznol M McDermott DF Kluger HM Carvajal RD Sharfman WH. Survival, Durable Tumor Remission, and Long-Term Safety in Patients With Advanced Melanoma Receiving Nivolumab. J Clin Oncol (2014) 32(10):1020–30. 10.1200/JCO.2013.53.0105 PMC4811023 24590637

[7] Topalian SL Hodi FS Brahmer JR Gettinger SN Smith DC McDermott DF. Safety, Activity, and Immune Correlates of Anti-PD-1 Antibody in Cancer. N Engl J Med (2012) 366(26):2443–54. 10.1056/NEJMoa1200690 PMC3544539 22658127

[8] Tran TT Jilaveanu LB Omuro A Chiang VL Huttner A Kluger HM. Complications Associated With Immunotherapy for Brain Metastases. Curr Opin Neurol (2019) 32(6):907–16. 10.1097/WCO.0000000000000756 PMC7398556 31577604

[9] Decazes P Bohn P. Immunotherapy by Immune Checkpoint Inhibitors and Nuclear Medicine Imaging: Current and Future Applications. Cancers (Basel) (2020) 12(2):371. 10.3390/cancers12020371 PMC7072145 32041105

[10] Ben-Haim S Ell P. 18f-Fdg PET and PET/CT in the Evaluation of Cancer Treatment Response. J Nucl Med (2009) 50(1):88–99. 10.2967/jnumed.108.054205 19139187

[11] Laing RE Nair-Gill E Witte ON Radu CG. Visualizing Cancer and Immune Cell Function With Metabolic Positron Emission Tomography. Curr Opin Genet Dev (2010) 20(1):100–5. 10.1016/j.gde.2009.10.008 PMC3799818 19931447

[12] Treglia G. Diagnostic Performance of (18)F-FDG PET/CT in Infectious and Inflammatory Diseases According to Published Meta-Analyses. Contrast Media Mol Imaging (2019) 2019:3018349. 10.1155/2019/3018349 31427907 PMC6683817

[13] Jamar F Buscombe J Chiti A Christian PE Delbeke D Donohoe KJ. EANM/SNMMI Guideline for 18F-FDG Use in Inflammation and Infection. J Nucl Med (2013) 54(4):647–58. 10.2967/jnumed.112.112524 23359660

[14] Tomita M Suzuki M Kono Y Nakajima K Matsuda T Kuge Y. Influence on [(18)F]FDG Uptake by Cancer Cells After Anti-PD-1 Therapy in an Enforced-Immune Activated Mouse Tumor. EJNMMI Res (2020) 10(1):24. 10.1186/s13550-020-0608-4 32189078 PMC7080890

[15] Lee S Choi S Kim SY Yun MJ Kim HI. Potential Utility of FDG Pet-CT as a non-Invasive Tool for Monitoring Local Immune Responses. J Gastric Cancer (2017) 17(4):384–93. 10.5230/jgc.2017.17.e43 PMC5746659 29302378

[16] Shu CJ Guo S Kim YJ Shelly SM Nijagal A Ray P. Visualization of a Primary Anti-Tumor Immune Response by Positron Emission Tomography. Proc Natl Acad Sci USA (2005) 102(48):17412–7. 10.1073/pnas.0508698102 PMC1283986 16293690

[17] Mazzaschi G Madeddu D Falco A Bocchialini G Goldoni M Sogni F. Low PD-1 Expression in Cytotoxic Cd8(+) Tumor-Infiltrating Lymphocytes Confers an Immune-Privileged Tissue Microenvironment in NSCLC With a Prognostic and Predictive Value. Clin Cancer Res (2018) 24(2):407–19. 10.1158/1078-0432.CCR-17-2156 29074606

[18] Tumeh PC Harview CL Yearley JH Shintaku IP Taylor EJ Robert L. PD-1 Blockade Induces Responses by Inhibiting Adaptive Immune Resistance. Nature (2014) 515(7528):568–71. 10.1038/nature13954 PMC4246418 25428505

[19] Horne ZD Jack R Gray ZT Siegfried JM Wilson DO Yousem SA. Increased Levels of Tumor-Infiltrating Lymphocytes are Associated With Improved Recurrence-Free Survival in Stage 1A non-Small-Cell Lung Cancer. J Surg Res (2011) 171(1):1–5. 10.1016/j.jss.2011.03.068 21571304

[20] Kluger HM Zito CR Barr ML Baine MK Chiang VL Sznol M. Characterization of PD-L1 Expression and Associated T-Cell Infiltrates in Metastatic Melanoma Samples From Variable Anatomic Sites. Clin Cancer Res (2015) 21(13):3052–60. 10.1158/1078-0432.CCR-14-3073 PMC4490112 25788491

[21] Murakami W Tozaki M Sasaki M Hida AI Ohi Y Kubota K. Correlation Between (18)F-FDG Uptake on PET/MRI and the Level of Tumor-Infiltrating Lymphocytes (Tils) in Triple-Negative and HER2-Positive Breast Cancer. Eur J Radiol (2020) 123:108773. 10.1016/j.ejrad.2019.108773 31918248

[22] Hirakata T Fujii T Kurozumi S Katayama A Honda C Yanai K. FDG Uptake Reflects Breast Cancer Immunological Features: The PD-L1 Expression and Degree of Tils in Primary Breast Cancer. Breast Cancer Res Treat (2020) 181(2):331–8. 10.1007/s10549-020-05619-0 32253685

[23] Wang Y Zhao N Wu Z Pan N Shen X Liu T. New Insight on the Correlation of Metabolic Status on (18)F-FDG PET/CT With Immune Marker Expression in Patients With Non-Small Cell Lung Cancer. Eur J Nucl Med Mol Imaging (2020) 47(5):1127–36. 10.1007/s00259-019-04500-7 31502013

[24] Lopci E Toschi L Grizzi F Rahal D Olivari L Castino GF. Correlation of Metabolic Information on FDG-PET With Tissue Expression of Immune Markers in Patients With non-Small Cell Lung Cancer (NSCLC) Who are Candidates for Upfront Surgery. Eur J Nucl Med Mol Imaging (2016) 43(11):1954–61. 10.1007/s00259-016-3425-2 27251642

[25] Cacho-Diaz B Garcia-Botello DR Wegman-Ostrosky T Reyes-Soto G Ortiz-Sanchez E Herrera-Montalvo LA. Tumor Microenvironment Differences Between Primary Tumor and Brain Metastases. J Transl Med (2020) 18(1):1. 10.1186/s12967-019-02189-8 31900168 PMC6941297

[26] Galldiks N Langen KJ Albert NL Chamberlain M Soffietti R Kim MM. PET Imaging in Patients With Brain Metastasis-Report of the RANO/PET Group. Neuro Oncol (2019) 21(5):585–95. 10.1093/neuonc/noz003 PMC6502495 30615138

[27] Das K Mittal BR Vasistha RK Singh P Mathuriya SN. Role of (18)F-Fluorodeoxyglucose Positron Emission Tomography Scan in Differentiating Enhancing Brain Tumors. Indian J Nucl Med (2011) 26(4):171–6. 10.4103/0972-3919.106698 PMC3613621 23559710

[28] Utriainen M Metsahonkala L Salmi TT Utriainen T Kalimo H Pihko H. Metabolic Characterization of Childhood Brain Tumors: Comparison of 18F-Fluorodeoxyglucose and 11C-Methionine Positron Emission Tomography. Cancer (2002) 95(6):1376–86. 10.1002/cncr.10798 12216107

[29] Kosaka N Tsuchida T Uematsu H Kimura H Okazawa H Itoh H. 18f-Fdg PET of Common Enhancing Malignant Brain Tumors. AJR Am J Roentgenol (2008) 190(6):W365–9. 10.2214/AJR.07.2660 18492879

[30] Akoglu H. User’s Guide to Correlation Coefficients. Turk J Emerg Med (2018) 18(3):91–3. 10.1016/j.tjem.2018.08.001 PMC6107969 30191186

[31] Di Giacomo AM Valente M Cerase A Lofiego MF Piazzini F Calabro L. Immunotherapy of Brain Metastases: Breaking a “Dogma”. J Exp Clin Cancer Res (2019) 38(1):419. 10.1186/s13046-019-1426-2 31623643 PMC6798349

[32] Quail DF Joyce JA. The Microenvironmental Landscape of Brain Tumors. Cancer Cell (2017) 31(3):326–41. 10.1016/j.ccell.2017.02.009 PMC5424263 28292436

[33] Berti V Mosconi L Pupi A. Brain: Normal Variations and Benign Findings in Fluorodeoxyglucose-PET/Computed Tomography Imaging. PET Clin (2014) 9(2):129–40. 10.1016/j.cpet.2013.10.006 PMC3998066 24772054

[34] Juhasz C Dwivedi S Kamson DO Michelhaugh SK Mittal S. Comparison of Amino Acid Positron Emission Tomographic Radiotracers for Molecular Imaging of Primary and Metastatic Brain Tumors. Mol Imaging (2014) 13:10.2310/7290.2014.00015. 10.2310/7290.2014.00015 PMC4199087 24825818

[35] Kasahara N Kaira K Yamaguchi K Masubuchi H Tsurumaki H Hara K. Fluorodeoxyglucose Uptake is Associated With Low Tumor-Infiltrating Lymphocyte Levels in Patients With Small Cell Lung Cancer. Lung Cancer (2019) 134:180–6. 10.1016/j.lungcan.2019.06.009 31319979

[36] Herbst RS Soria JC Kowanetz M Fine GD Hamid O Gordon MS. Predictive Correlates of Response to the Anti-PD-L1 Antibody MPDL3280A in Cancer Patients. Nature (2014) 515(7528):563–7. 10.1038/nature14011 PMC4836193 25428504

[37] Rossi S Toschi L Castello A Grizzi F Mansi L Lopci E. Clinical Characteristics of Patient Selection and Imaging Predictors of Outcome in Solid Tumors Treated With Checkpoint-Inhibitors. Eur J Nucl Med Mol Imaging (2017) 44(13):2310–25. 10.1007/s00259-017-3802-5 28815334

[38] Chang CH Qiu J O’Sullivan D Buck MD Noguchi T Curtis JD. Metabolic Competition in the Tumor Microenvironment Is a Driver of Cancer Progression. Cell (2015) 162(6):1229–41. 10.1016/j.cell.2015.08.016 PMC4864363 26321679

[39] Sampson JH Gunn MD Fecci PE Ashley DM. Brain Immunology and Immunotherapy in Brain Tumours. Nat Rev Cancer (2020) 20(1):12–25. 10.1038/s41568-019-0224-7 31806885 PMC7327710

[40] Farber SH Tsvankin V Narloch JL Kim GJ Salama AK Vlahovic G. Embracing Rejection: Immunologic Trends in Brain Metastasis. Oncoimmunology (2016) 5(7):e1172153. 10.1080/2162402X.2016.1172153 27622023 PMC5006920

[41] You H Baluszek S Kaminska B. Immune Microenvironment of Brain Metastases-are Microglia and Other Brain Macrophages Little Helpers? Front Immunol (2019) 10:1941. 10.3389/fimmu.2019.01941 31481958 PMC6710386

[42] Kajary K Lengyel Z Tokes AM Kulka J Dank M Tokes T. Dynamic FDG-PET/CT in the Initial Staging of Primary Breast Cancer: Clinicopathological Correlations. Pathol Oncol Res (2020) 26(2):997–1006. 10.1007/s12253-019-00641-0 30941738 PMC7242263

[43] Sasada S Shiroma N Goda N Kajitani K Emi A Masumoto N. The Relationship Between Ring-Type Dedicated Breast PET and Immune Microenvironment in Early Breast Cancer. Breast Cancer Res Treat (2019) 177(3):651–7. 10.1007/s10549-019-05339-0 31267329

[44] Galdiero MR Bonavita E Barajon I Garlanda C Mantovani A Jaillon S. Tumor Associated Macrophages and Neutrophils in Cancer. Immunobiology (2013) 218(11):1402–10. 10.1016/j.imbio.2013.06.003 23891329

[45] Pukrop T Dehghani F Chuang HN Lohaus R Bayanga K Heermann S. Microglia Promote Colonization of Brain Tissue by Breast Cancer Cells in a Wnt-Dependent Way. Glia (2010) 58(12):1477–89. 10.1002/glia.21022 20549749

[46] Laoui D Movahedi K Van Overmeire E Van den Bossche J Schouppe E Mommer C. Tumor-Associated Macrophages in Breast Cancer: Distinct Subsets, Distinct Functions. Int J Dev Biol (2011) 55(7-9):861–7. 10.1387/ijdb.113371dl 22161841

[47] Park SG Lee JH Lee WA Han KM. Biologic Correlation Between Glucose Transporters, Hexokinase-II, Ki-67 and FDG Uptake in Malignant Melanoma. Nucl Med Biol (2012) 39(8):1167–72. 10.1016/j.nucmedbio.2012.07.003 22901702

[48] Tohma T Okazumi S Makino H Cho A Mochiduki R Shuto K. Relationship Between Glucose Transporter, Hexokinase and FDG-PET in Esophageal Cancer. Hepatogastroenterology (2005) 52(62):486–90. 15816463

[49] Izuishi K Yamamoto Y Sano T Takebayashi R Nishiyama Y Mori H. Molecular Mechanism Underlying the Detection of Colorectal Cancer by 18F-2-Fluoro-2-Deoxy-D-Glucose Positron Emission Tomography. J Gastrointest Surg (2012) 16(2):394–400. 10.1007/s11605-011-1727-z 22065316

[50] Mamede M Higashi T Kitaichi M Ishizu K Ishimori T Nakamoto Y. [18F]FDG Uptake and PCNA, Glut-1, and Hexokinase-II Expressions in Cancers and Inflammatory Lesions of the Lung. Neoplasia (2005) 7(4):369–79. 10.1593/neo.04577 PMC1501150 15967114

[51] Deng SM Zhang W Zhang B Chen YY Li JH Wu YW. Correlation Between the Uptake of 18F-Fluorodeoxyglucose (18f-FDG) and the Expression of Proliferation-Associated Antigen Ki-67 in Cancer Patients: A Meta-Analysis. PloS One (2015) 10(6):e0129028. 10.1371/journal.pone.0129028 26038827 PMC4454667

[52] Szekely B Bossuyt V Li X Wali VB Patwardhan GA Frederick C. Immunological Differences Between Primary and Metastatic Breast Cancer. Ann Oncol (2018) 29(11):2232–9. 10.1093/annonc/mdy399 30203045

