# Supplementary material for: Correlation Between 18F-FDG Uptake and Immune Cell Infiltration in Metastatic Brain Lesions
Source: Front Oncol. 2021 Jun 24;11:618705. doi: 10.3389/fonc.2021.618705 (PMC8266210; doi:10.3389/fonc.2021.618705)
Supplement: Supplementary file 1 [file Table_1.docx]

**Supplementary Table 1**. Clinical characteristics in 34 patients with brain metastasis

| Case number | Sex | Age (years) | Primary site of cancer | Histologic type of primary cancer | Size of metastatic brain lesion (cm) | Size of volume of interest (cm^3^) | Maximum ^18^F-FDG uptake ratio | Mean ^18^F-FDG uptake ratio | Expression grade of GLUT1 | Expression grade of HK2 | Expression grade of MPO | Expression grade of CD68 | Expression grade of CD163 | Expression grade of CD3 | Expression grade of CD8 | Ki-67 proliferation index (%) |
| --- | --- | --- | --- | --- | --- | --- | --- | --- | --- | --- | --- | --- | --- | --- | --- | --- |
| 1 | M | 57 | colon | Adenocarcinoma | 4.68 | 26.01 | 4.01 | 2.22 | 2 | 2 | 3 | 2 | 3 | 2 | 1 | 54.5 |
| 2 | M | 59 | lung | Adenocarcinoma | 3.83 | 17.88 | 2.37 | 1.64 | 2 | 3 | 3 | 4 | 4 | 2 | 1 | 19.6 |
| 3 | M | 71 | lung | Adenocarcinoma | 2.99 | 10.65 | 1.60 | 0.84 | 4 | 4 | 3 | 3 | 3 | 2 | 1 | 33.3 |
| 4 | F | 63 | breast | Adenocarcinoma | 3.57 | 15.45 | 3.94 | 2.36 | 1 | 4 | 1 | 2 | 2 | 2 | 1 | 1.1 |
| 5 | F | 54 | breast | Adenocarcinoma | 4.45 | 21.61 | 4.29 | 2.90 | 1 | 4 | 1 | 2 | 2 | 1 | 1 | 7.7 |
| 6 | M | 66 | stomach | Adenocarcinoma | 2.39 | 16.33 | 3.68 | 1.75 | 2 | 2 | 3 | 3 | 2 | 1 | 1 | 96.1 |
| 7 | F | 60 | ovary | Adenocarcinoma | 1.66 | 1.51 | 2.01 | 1.55 | 2 | 2 | 1 | 2 | 2 | 2 | 1 | 39.8 |
| 8 | M | 79 | lung | Adenocarcinoma | 4.36 | 19.83 | 3.89 | 1.55 | 4 | 4 | 3 | 2 | 2 | 1 | 1 | 72.8 |
| 9 | F | 54 | breast | Adenocarcinoma | 4.6 | 36.19 | 2.80 | 1.20 | 1 | 4 | 1 | 1 | 1 | 1 | 1 | 1.7 |
| 10 | M | 68 | rectum | Adenocarcinoma | 1.92 | 5.86 | 1.49 | 0.94 | 3 | 3 | 2 | 3 | 3 | 2 | 1 | 77.0 |
| 11 | M | 77 | liver | Adenocarcinoma | 3.24 | 16.59 | 1.97 | 1.00 | 2 | 1 | 4 | 2 | 3 | 2 | 2 | 41.3 |
| 12 | F | 64 | breast | Adenocarcinoma | 1.89 | 1.19 | 2.63 | 2.06 | 1 | 4 | 1 | 1 | 1 | 1 | 1 | 14.6 |
| 13 | F | 80 | breast | Adenocarcinoma | 2.33 | 2.53 | 2.32 | 2.03 | 3 | 3 | 2 | 1 | 1 | 1 | 1 | 65.4 |
| 14 | F | 77 | lung | Adenocarcinoma | 2.64 | 6.25 | 4.01 | 2.34 | 4 | 3 | 1 | 1 | 2 | 1 | 1 | 55.0 |
| 15 | M | 69 | stomach | Adenocarcinoma | 3.11 | 8.94 | 3.16 | 1.08 | 3 | 2 | 2 | 1 | 1 | 1 | 1 | 81.2 |
| 16 | F | 55 | lung | Adenocarcinoma | 1.97 | 2.55 | 2.05 | 1.26 | 2 | 3 | 2 | 4 | 4 | 4 | 3 | 29.4 |
| 17 | F | 64 | breast | Adenocarcinoma | 3.12 | 15.25 | 1.83 | 0.53 | 2 | 4 | 1 | 1 | 1 | 1 | 1 | 28.3 |
| 18 | M | 59 | lung | Squamous cell carcinoma | 4.05 | 26.36 | 2.08 | 1.18 | 3 | 1 | 4 | 4 | 4 | 2 | 1 | 39.4 |
| 19 | M | 64 | esophagus | Squamous cell carcinoma | 2.44 | 3.88 | 2.17 | 1.42 | 4 | 2 | 2 | 1 | 1 | 1 | 1 | 20.4 |
| 20 | M | 72 | colon | Adenocarcinoma | 2.87 | 7.9 | 2.98 | 1.98 | 3 | 2 | 2 | 3 | 3 | 1 | 1 | 68.5 |
| 21 | F | 69 | lung | Adenocarcinoma | 3.74 | 17.25 | 1.73 | 1.09 | 4 | 4 | 1 | 1 | 2 | 1 | 1 | 40.6 |
| 22 | M | 55 | lung | Adenocarcinoma | 2.35 | 1.72 | 2.02 | 1.40 | 1 | 1 | 2 | 3 | 4 | 3 | 3 | 0.3 |
| 23 | F | 62 | breast | Adenocarcinoma | 4.14 | 35.29 | 4.63 | 3.05 | 1 | 3 | 1 | 2 | 2 | 1 | 1 | 14.1 |
| 24 | M | 82 | lung | Squamous cell carcinoma | 3.66 | 12.11 | 1.85 | 0.92 | 2 | 2 | 2 | 2 | 2 | 1 | 1 | 56.3 |
| 25 | M | 59 | lung | Adenocarcinoma | 3.89 | 17.26 | 3.16 | 1.42 | 1 | 1 | 3 | 4 | 3 | 2 | 1 | 8.4 |
| 26 | M | 71 | lung | Adenocarcinoma | 3.06 | 11.75 | 4.06 | 1.28 | 3 | 4 | 3 | 3 | 3 | 2 | 2 | 14.6 |
| 27 | F | 47 | breast | Adenocarcinoma | 2.05 | 6.57 | 2.68 | 1.84 | 1 | 2 | 1 | 1 | 2 | 1 | 1 | 16.8 |
| 28 | F | 66 | breast | Adenocarcinoma | 4.31 | 29.31 | 4.22 | 2.72 | 3 | 2 | 1 | 2 | 2 | 2 | 2 | 7.1 |
| 29 | F | 52 | breast | Adenocarcinoma | 2.61 | 4.1 | 1.95 | 1.16 | 2 | 3 | 3 | 1 | 2 | 2 | 1 | 25.0 |
| 30 | M | 47 | lung | Large cell neuroendocrine carcinoma | 1.53 | 1.12 | 4.38 | 3.11 | 4 | 1 | 3 | 4 | 4 | 2 | 2 | 36.9 |
| 31 | M | 54 | lung | Adenocarcinoma | 1.88 | 3.47 | 3.72 | 1.61 | 4 | 3 | 2 | 1 | 1 | 1 | 1 | 29.5 |
| 32 | M | 56 | prostate | Adenocarcinoma | 3.25 | 6.19 | 7.63 | 3.28 | 2 | 4 | 1 | 1 | 1 | 1 | 1 | 1.1 |
| 33 | M | 52 | lung | Adenocarcinoma | 2.81 | 10.86 | 2.72 | 1.56 | 3 | 3 | 2 | 2 | 3 | 2 | 2 | 14.2 |
| 34 | M | 82 | lung | Small cell carcinoma | 4.16 | 25.1 | 2.67 | 1.52 | 3 | 1 | 1 | 1 | 1 | 1 | 1 | 78.6 |

GLUT1, Glucose transporter 1; HK2, Hexokinase 2; MPO, Myeloperoxidase, marker for neutrophils; CD3/CD8, Marker for T cells; CD68/CD163, Marker for macrophages

**Supplementary Table 2**. Correlations among the expression of GLUT1, HK2, and Ki-67 and immune cell markers

|  | | p-value (*rho* [95% CI]) versus | | | | | | | |
| --- | --- | --- | --- | --- | --- | --- | --- | --- | --- |
| Primary cancer |  | MPO | CD68 | CD163 | CD3 | CD8 | GLUT1 | HK2 | Ki-67 |
| Total | MPO | - | < 0.001^*^ (0.58 [0.31 to 0.77]) | < 0.001^*^ (0.59 [0.32 to 0.77]) | 0.006^*^ (0.46 [0.14 to 0.69]) | 0.213 (0.29 [-0.13 to 0.52]) | 0.219 (0.22 [-0.13 to 0.52]) | 0.524 (0.11 [-0.23 to 0.44]) | 0.076 (0.31 [-0.03 to 0.59]) |
|  | CD68 | < 0.001^*^ (0.58 [0.31 to 0.77]) | - | < 0.001^*^ (0.88 [0.77 to 0.94]) | < 0.001^*^ (0.67 [0.43 to 0.82]) | 0.019^*^ (0.40 [0.07 to 0.65]) | 0.911 (-0.02 [-0.36 to 0.32]) | 0.078 (0.11 [-0.23 to 0.44]) | 0.911 (-0.02 [-0.36 to 0.32]) |
|  | CD163 | < 0.001^*^ (0.59 [0.32 to 0.77]) | < 0.001^*^ (0.88 [0.77 to 0.94]) | - | < 0.001^*^ (0.78 [0.60 to 0.88]) | 0.002^*^ (0.52 [0.22 to 0.73]) | 0.830 (-0.04 [-0.37 to 0.30]) | 0.056 (-0.33 [-0.60 to 0.01]) | 0.814 (-0.04 [-0.38 to 0.30]) |
|  | CD3 | 0.006^*^ (0.46 [0.14 to 0.69]) | < 0.001^*^ (0.67 [0.43 to 0.82]) | < 0.001^*^ (0.78 [0.60 to 0.88]) | - | < 0.001^*^ (0.62 [0.36 to 0.79]) | 0.804 (-0.04 [-0.38 to 0.30]) | 0.131 (-0.27 [-0.55 to 0.08]) | 0.138 (-0.26 [-0.55 to 0.09]) |
|  | CD8 | 0.213 (0.29 [-0.13 to 0.52]) | 0.019^*^ (0.40 [0.07 to 0.65]) | 0.002^*^ (0.52 [0.22 to 0.73]) | < 0.001^*^ (0.62 [0.36 to 0.79]) | - | 0.750 (0.06 [-0.29 to 0.39) | 0.164 (-0.24 [-0.54 to 0.10]) | 0.110 (-0.28 [-0.56 to 0.07]) |
|  | GLUT1 | 0.219 (0.22 [-0.13 to 0.52]) | 0.911 (-0.02 [-0.36 to 0.32]) | 0.830 (-0.04 [-0.37 to 0.30]) | 0.804 (-0.04 [-0.38 to 0.30]) | 0.750 (0.06 [-0.29 to 0.39]) | - | 0.907 (0.02 [-0.32 to 0.36]) | 0.001^*^ (0.53 [0.23 to 0.73]) |
|  | HK2 | 0.524 (0.11 [-0.23 to 0.44]) | 0.078 (0.11 [-0.23 to 0.44]) | 0.056 (-0.33 [-0.60 to 0.01]) | 0.131 (-0.27 [-0.55 to 0.08]) | 0.164 (-0.24 [-0.54 to 0.10]) | 0.907 (0.02 [-0.32 to 0.36]) | - | 0.113 (-0.28 [-0.56 to 0.07]) |
|  | Ki-67 | 0.076 (0.31 [-0.03 to 0.59]) | 0.911 (-0.02 [-0.36 ^to^ 0.32]) | 0.814 (-0.04 [-0.38 to 0.30]) | 0.138 (-0.26 [-0.55 to 0.09]) | 0.110 (-0.28 [-0.56 to 0.07]) | 0.001^*^ (0.53 [0.23 to 0.73]) | 0.113 (-0.28 [-0.56 to 0.07]) | - |
| Lung | MPO | - | < 0.001^*^ (0.81 [0.49 to 0.94]) | 0.014^*^ (0.64 [0.17 to 0.87]) | 0.054 (0.53 [-0.01 to 0.83]) | 0.890 (0.04 [-0.50 to 0.56]) | 0.321 (-0.29 [-0.71 to 0.29]) | 0.636 (-0.14 [-0.62 to 0.42]) | 0.162 (-0.40 [-0.77 to 0.17]) |
|  | CD68 | < 0.001^*^ (0.81 [0.49 to 0.94]) | - | < 0.001^*^ (0.88 [0.66 to 0.96]) | < 0.001^*^ (0.79 [0.44 to 0.93]) | 0.257 (0.33 [-0.25 to 0.73]) | 0.610 (-0.09 [-0.42 to 0.26]) | 0.284 (-0.31 [-0.72 to 0.27]) | 0.09 (-0.46 [-0.80 to 0.09]) |
|  | CD163 | 0.014^*^ (0.64 [0.17 to 0.87]) | < 0.001^*^ (0.88 [0.66 to 0.96]) | - | < 0.001^*^ (0.88 [0.66 to 0.96]) | 0.039^*^ (0.56 [0.04 to 0.84]) | 0.092 (-0.47 [-0.80 to 0.08]) | 0.422 (-0.23 [-0.68 to 0.34]) | 0.060 (-0.52 [-0.82 to 0.02]) |
|  | CD3 | 0.054 (0.53 [-0.01 to 0.83]) | < 0.001^*^ (0.79 [0.44 to 0.93]) | < 0.001^*^ (0.88 [0.66 to 0.96]) | - | < 0.003^*^ (0.73 [0.33 to 0.91]) | 0.053 (-0.53 [-0.83 to 0.01]) | 0.660 (-0.13 [-0.62 to 0.43]) | 0.003^*^ (-0.73 [-0.91 to -0.32]) |
|  | CD8 | 0.890 (0.04 [-0.50 to 0.56]) | 0.257 (0.33 [-0.25 to 0.73]) | 0.039^*^ (0.56 [0.04 to 0.84]) | < 0.003^*^ (0.73 [0.33 to 0.91]) | - | 0.421 (-0.23 [-0.68 to 0.34) | 0.847 (-0.06 [-0.57 to 0.49]) | 0.041^*^ (-0.55 [-0.84 to -0.07]) |
|  | GLUT1 | 0.321 (-0.29 [-0.71 to 0.29]) | 0.610 (-0.09 [-0.42 to 0.26]) | 0.092 (-0.47 [-0.80 to 0.08]) | 0.053 (-0.53 [-0.83 to 0.01]) | 0.421 (-0.23 [-0.68 to 0.34) | - | 0.060 (0.52 [-0.02 to 0.82]) | 0.093 (0.47 [-0.09 to 0.80]) |
|  | HK2 | 0.636 (-0.14 [-0.62 to 0.42]) | 0.284 (-0.31 [-0.72 to 0.27]) | 0.422 (-0.23 [-0.68 to 0.34]) | 0.660 (-0.13 [-0.62 to 0.43]) | 0.847 (-0.06 [-0.57 to 0.49]) | 0.060 (0.52 [-0.02 to 0.82]) | - | 0.931 (-0.03 [-0.55 to 0.51]) |
|  | Ki-67 | 0.162 (-0.40 [-0.77 to 0.17]) | 0.090 (-0.46 [-0.80 to 0.09]) | 0.060 (-0.52 [-0.82 to 0.02]) | 0.003^*^ (-0.73 [-0.91 to -0.32]) | 0.041^*^ (-0.55 [-0.84 to -0.07]) | 0.093 (0.47 [-0.09 to 0.80]) | 0.931 (-0.03 [-0.55 to 0.51]) | - |
| Breast | MPO | - | 0.245 (-0.41 [-0.83 to 0.30]) | 0.889 (-0.05 [-0.66 to 0.60]) | 0.449 (0.27 [-0.43 to 0.77]) | 0.648 (-0.17 [-0.72 to 0.52]) | 0.086 (0.57 [-0.09 to 0.88]) | 0.430 (-0.28 [-0.77 to 0.42]) | 0.073 (0.59 [-0.07 to 0.89]) |
|  | CD68 | 0.245 (-0.41 [-0.83 to 0.30]) | - | < 0.035^*^ (0.67 [0.06 to 0.91]) | 0.312 (0.36 [-0.35 to 0.81]) | 0.242 (0.41 [-0.30 to 0.83]) | 0.656 (-0.16 [-0.72 to 0.52]) | 0.916 (-0.04 [-0.65 to 0.61]) | 0.047^*^ (-0.64 [-0.91 to -0.02]) |
|  | CD163 | 0.889 (-0.05 [-0.66 to 0.60]) | < 0.035^*^ (0.67 [0.06 to 0.91]) | - | 0.111 (0.53 [-0.14 to 0.87]) | 0.272 (0.45 [-0.43 to 0.77]) | 0.656 (-0.16 [-0.72 to 0.52]) | 0.178 (-0.46 [-0.85 to 0.24]) | 0.314 (-0.36 [-0.81 to 0.35]) |
|  | CD3 | 0.449 (0.27 [-0.43 to 0.77]) | 0.312 (0.36 [-0.35 to 0.81]) | 0.111 (0.53 [-0.14 to 0.87]) | - | 0.242 (0.41 [-0.30 to 0.83]) | 0.329 (0.35 [-0.36 to 0.8 0]) | 0.491 (-0.25 [-0.76 to 0.45]) | 0.334 (-0.34 [-0.80 to 0.37]) |
|  | CD8 | 0.648 (-0.17 [-0.72 to 0.52]) | 0.242 (0.41 [-0.30 to 0.83]) | 0.272 (0.45 [-0.43 to 0.77]) | 0.242 (0.41 [-0.30 to 0.83]) | - | 0.118 (0.53 [-0.15 to 0.87]) | 0.138 (-0.50 [-0.86 to 0.18]) | 0.416 (-0.29 [-0.78 to 0.42]) |
|  | GLUT1 | 0.086 (0.57 [-0.09 to 0.88]) | 0.656 (-0.16 [-0.72 to 0.52]) | 0.656 (-0.16 [-0.72 to 0.52]) | 0.329 (0.35 [-0.36 to 0.8 0]) | 0.118 (0.53 [-0.15 to 0.87]) | - | 0.211 (-0.43 [-0.84 to 0.27]) | 0.145 (0.50 [-0.20 to 0.86]) |
|  | HK2 | 0.430 (-0.28 [-0.77 to 0.42]) | 0.916 (-0.04 [-0.65 to 0.61]) | 0.178 (-0.46 [-0.85 to 0.24]) | 0.491 (-0.25 [-0.76 to 0.45]) | 0.138 (-0.50 [-0.86 to 0.18]) | 0.211 (-0.43 [-0.84 to 0.27]) | - | 0.474 (-0.26 [-0.76 to 0.45]) |
|  | Ki-67 | 0.073 (0.59 [-0.07 to 0.89]) | 0.047^*^ (-0.64 [-0.91 to -0.02]) | 0.314 (-0.36 [-0.81 to 0.35]) | 0.334 (-0.34 [-0.80 to 0.37]) | 0.416 (-0.29 [-0.78 to 0.42]) | 0.145 (0.50 [-0.20 to 0.86]) | 0.474 (-0.26 [-0.76 to 0.45]) | - |
| GI tract and others | MPO | - | 0.389 (0.31 [-0.41 to 0.79]) | 0.162 (0.48 [-0.22 to 0.85]) | 0.681 (0.15 [-0.53 to 0.71]) | 0.101 (0.55 [-0.12 to 0.88]) | 0.932 (-0.03 [-0.65 to 0.61]) | 0.273 (-0.39 [-0.82 to 0.32]) | 0.243 (0.41 [-0.03 to 0.83]) |
|  | CD68 | 0.389 (0.31 [-0.41 to 0.79]) | - | 0.010^*^ (0.76 [0.25 to 0.94]) | 0.462 (0.26 [-0.44 to 0.77]) | 1.00 (0.00 [-0.63 to 0.63]) | 0.737 (-0.12 [-0.70 to 0.55]) | 0.834 (-0.08 [-0.67 to 0.58]) | 0.108 (0.54 [-0.14 to 0.87]) |
|  | CD163 | 0.162 (0.48 [-0.22 to 0.85]) | 0.010^*^ (0.76 [0.25 to 0.94]) | - | 0.046^*^ (0.64 [0.02 to 0.91]) | 0.294 (0.37 [-0.34 to 0.81]) | 0.536 (-0.22 [-0.75 to 0.47]) | 0.416 (-0.29 [-0.78 to 0.42]) | 0.545 (0.22 [-0.48 to 0.75]) |
|  | CD3 | 0.681 (0.15 [-0.53 to 0.71]) | 0.462 (0.26 [-0.44 to 0.77]) | 0.046^*^ (0.64 [0.02 to 0.91]) | - | 0.242 (0.41 [-0.30 to 0.83]) | 0.178 (-0.46 [-0.85 to 0.24]) | 0.365 (-0.32 [-0.79 to 0.39]) | 0.695 (-0.14 [-0.71 to 0.54]) |
|  | CD8 | 0.101 (0.55 [-0.12 to 0.88]) | 1.00 (0.00 [-0.63 to 0.63]) | 0.294 (0.37 [-0.34 to 0.81]) | 0.242 (0.41 [-0.30 to 0.83]) | - | 0.375 (-0.32 [-0.79 to 0.39]) | 0.072 (-0.59 [-0.89 to 0.06]) | 0.630 (-0.17 -0.72o 0.51]) |
|  | GLUT1 | 0.932 (-0.03 [-0.65 to 0.61]) | 0.737 (-0.12 [-0.70 to 0.55]) | 0.536 (-0.22 [-0.75 to 0.47]) | 0.178 (-0.46 [-0.85 to 0.24]) | 0.375 (-0.32 [-0.79 to 0.39]) | - | 0.367 (0.32 [-0.39 to 0.79]) | 0.637 (0.17 [-0.51 to 0.72]) |
|  | HK2 | 0.273 (-0.39 [-0.82 to 0.32]) | 0.834 (-0.08 [-0.67 to 0.58]) | 0.416 (-0.29 [-0.78 to 0.42]) | 0.365 (-0.32 [-0.79 to 0.39]) | 0.072 (-0.59 [-0.89 to 0.06]) | 0.367 (0.32 [-0.39 to 0.79]) | - | 0.970 (-0.01 [-0.64 to 0.62]) |
|  | Ki-67 | 0.243 (0.41 [-0.03 to 0.83]) | 0.108 (0.54 [-0.14 to 0.87]) | 0.545 (0.22 [-0.48 to 0.75]) | 0.695 (-0.14 [-0.71 to 0.54]) | 0.630 (-0.17 -0.72o 0.51]) | 0.637 (0.17 [-0.51 to 0.72]) | 0.970 (-0.01 [-0.64 to 0.62]) | - |

^*^ p < 0.05: GI tract: Gastrointestinal tract; GLUT1, Glucose transporter 1; HK2, Hexokinase 2; MPO, Myeloperoxidase, marker for neutrophils; CD3/CD8, Marker for T cells; CD68/CD163, Marker for macrophages
